# Transcriptional Regulation of the Mitochondrial Citrate and Carnitine/Acylcarnitine Transporters: Two Genes Involved in Fatty Acid Biosynthesis and β-oxidation 

**DOI:** 10.3390/biology2010284

**Published:** 2013-01-29

**Authors:** Vito Iacobazzi, Vittoria Infantino, Ferdinando Palmieri

**Affiliations:** 1 Department of Biosciences, Biotechnology and Pharmacological Sciences, University of Bari, Via Orabona 4, 70125 Bari, Italy; E-Mails: vittoria.infantino@unibas.it (V.I.); ferdpalmieri@gmail.com (F.P.); 2 Center of Excellence in Comparative Genomics, University of Bari, via Orabona 4, 70125 Bari, Italy; 3 CNR Institute of Biomembranes and Bioenergetics, 70125 Bari, Italy; 4 Department of Sciences, University of Basilicata, 85100 Potenza, Italy

**Keywords:** citrate carrier, carnitine carrier, gene expression, mitochondrial transporters, transcriptional regulation

## Abstract

Transcriptional regulation of genes involved in fatty acid metabolism is considered the major long-term regulatory mechanism controlling lipid homeostasis. By means of this mechanism, transcription factors, nutrients, hormones and epigenetics control not only fatty acid metabolism, but also many metabolic pathways and cellular functions at the molecular level. The regulation of the expression of many genes at the level of their transcription has already been analyzed. This review focuses on the transcriptional control of two genes involved in fatty acid biosynthesis and oxidation: the citrate carrier (CIC) and the carnitine/ acylcarnitine/carrier (CAC), which are members of the mitochondrial carrier gene family, SLC25. The contribution of tissue-specific and less tissue-specific transcription factors in activating or repressing CIC and CAC gene expression is discussed. The interaction with drugs of some transcription factors, such as PPAR and FOXA1, and how this interaction can be an attractive therapeutic approach, has also been evaluated. Moreover, the mechanism by which the expression of the CIC and CAC genes is modulated by coordinated responses to hormonal and nutritional changes and to epigenetics is highlighted.

## 1. Introduction

The regulation of metabolism occurs through different types of mechanisms: short-term mechanisms involving allosteric control and post- transcriptional modifications, and long-term control by transcriptional regulation. Allosteric control triggers the binding of an activating/de-activating substrate to a key enzyme. The post-transcriptional modifications (phosphorylation, glycosylation, proteolytic cleavage, *etc*.) shift the equilibrium between the active and inactive forms of an enzyme. Longer time control involves transcriptional regulation that affects the expression levels of key proteins. It requires specific signals to be transduced to the nucleus where defined sets of genes are targeted. Thus, understanding the transcriptional control of metabolism requires the detailed knowledge of events upstream of transcriptional activity—*i.e.*, the molecular mechanisms by which transcriptional factors operate—and events downstream of transcriptional activity, which depend on the set of genes that are targeted and how further signals are generated to reach the dynamic equilibrium of homeostasis [1]. 

Virtually all transcription factor families are involved in gene regulation. Some of them play a crucial role in the control of gene expression involved in a specific pathway. For example, SREBPs, C/EBPs, and members of the nuclear receptor family are particularly active regulators of the genes involved in fatty acid and cholesterol biosynthesis [2,3,4]. Transcriptional regulation of the fatty acid β-oxidation pathway has been dominated by the major role of PPARα [5]. 

**Figure 1:**
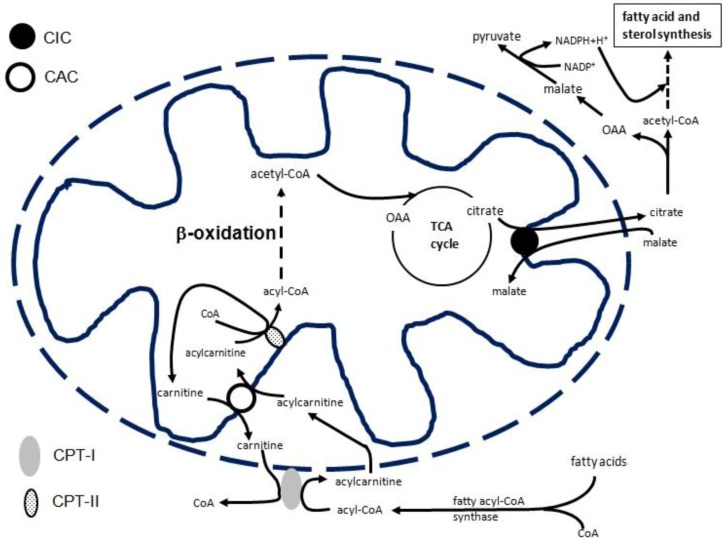
Role of CIC and CAC in fatty acid biosynthesis and β-oxidation, respectively Abbreviations: CPT-I, carnitine-palmitoyltransferase 1; CPT-II, carnitine-palmitoyltransferase 2; CIC, citrate carrier; CAC, carnitine/acylcarnitine carrier.

Metabolic pathways are often located in different cellular compartments and need to be linked through translocation of solutes across membranes. Mitochondrial carriers provide a link between mitochondria and cytosol by facilitating the flux of different metabolites, nucleotides, and coenzymes through the inner mitochondrial membrane [6,7,8]. Until now, 53 mitochondrial carriers have been identified on the human genome a nd more than half have been functionally characterized [6,7,8]. Although they play a crucial role in intermediary metabolism, only a few of them have been studied transcriptionally to some extent: the adenine nucleotide carrier [9,10,11,12,13], the uncoupling proteins [14,15,16,17,18,19], and the phosphate carrier [20] genes. Recently, the citrate carrier (CIC), encoded by SLC25A1, and the carnitine/acylcarnitine carrier (CAC), encoded by SLC25A20, have also been transcriptionally studied. CIC exports citrate from the mitochondrion to the cytosol, where it is cleaved by ATP-citrate lyase to oxaloacetate (OAA) and acetyl-CoA, which is used for fatty acid and sterol biosynthesis. OAA produced in the cytosol by citrate lyase is reduced to malate, which is converted to pyruvate via the malic enzyme with production of cytosolic NADPH plus H^+^ (necessary for fatty acid, and sterol synthesis) (Figure 1) [7]. As a component of the carnitine cycle, together with carnitine-palmitoyltransferase I and II (CPT-I and CPT-II), CAC catalyzes the transport of acylcarnitines (produced by CPT-1 as acyl-CoA) into mitochondria in exchange for free internal carnitine. In the mitochondrial matrix, CPT-II allows for transfer of the fatty acyl-units from carnitine to the matrix CoA, thus producing intramitochondrial acyl-CoAs which are oxidized by the enzymes of the β-oxidation pathway (Figure 1) [7]. Transcriptional regulation of the CIC and CAC genes is particularly important, since fatty acids affect a large number of cellular systems and functions. Furthermore, fatty acids are considered the major source of energy for the organism; as components of phospholipids, they play a structural role in cellular membranes and fatty acid-derived small bioactive molecules, such as arachidonic acid and other intracellular messengers, and have a crucial role in signaling pathways [21]. 

This review is focused on the molecular aspects of the transcriptional regulation of the CIC and CAC genes, mainly in humans. The role of transcriptional factors, hormonal and nutritional factors, and epigenetic mechanisms are highlighted in the context of fatty acid metabolism and tissue expression.

## 2. CIC Gene Transcriptional Regulation

The human CIC gene, localized on chromosome 22q11.21 [22], spans over 2.8 kb of human DNA and is divided into eight exons [23]. CIC mRNA and protein levels are high in liver, pancreas, and kidney, but low or absent in brain, heart, skeletal muscle, placenta, and lung [24].

The human CIC gene core promoter region lacks canonical TATA and CAAT boxes and presents the typical initiator element (Inr) containing the transcriptional start site, CCATAATT, identical to the consensus sequence Py-Py-A-N-T/A-PyPy. According to the database (http://dbtss.hgc.jp/index.html) of the transcriptional start site, adenine at position −89 bp, inside the Inr element, and the ATG codon upstream, the transcription start site is found in 16 of 20 5’-oligocapping clones. Furthermore, the sequence 5’-GGACC-3’ is located in a region from −33 to −38 bp upstream ATG, as is consistent with the consensus sequence of the downstream core promoter element (DPE). Moreover, the core promoter is embedded in a CpG island and shows the configuration of a typical CpG island promoter [25]. 

**Figure 2:**
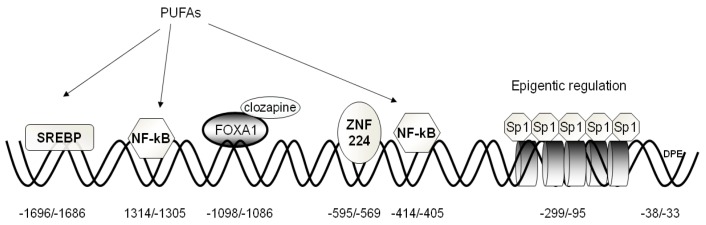
Structural organization of the human CIC gene proximal promoter. The basal elements (BRE and DPE), positive and negative responsive sequences, as well as their cognate transcription factors are indicated. Histones involved in epigenetic regulation are depicted as cylinders.

The 5’ regulatory region contains numerous binding sites for a variety of transcription factors acting as activators or repressors (Figure 2). The following have been tested functionally: Sp1, FOXA1, SREBP-1, NF-kB, and ZNF224. In the following sections, we will attempt to summarize the results of these studies and describe the regulatory roles that different transcription factors play in CIC gene expression.

### 2.1. Activators

*Sp1.* Seven consensus Sp1-binding sequences embedded in a CpG island, located between −299 and 10 bp (Figure 2), have been found in the CIC gene promoter. Five of them, located at positions −299/290, −206/−197, −162/−153, −137/−128, and 109/−100 bp, are functionally active, as demonstrated by EMSA and transfection experiments [26]. Sp1-binding activity depends on the methylation status of the Sp1-binding sequence. Transfection experiments performed in HepG2 cells with a construct containing the methylated CpG region demonstrate a marked decrease in luciferase (LUC) activity as compared to the activity of cells transfected with unmethylated construct. Moreover, ChIP analysis revealed that Sp1 binds to the CIC proximal promoter only in HepG2, whereas no binding was observed in SK-N-SH cells [26], in agreement with the active role performed by CIC in liver. However, since Sp1 is a universally expressed transcription factor [27], it is likely that it acts in concert with other transcription factors to activate CIC gene expression.

*FOXA1.* An active FOXA site is present at position –1098/–1088 bp that acts as a strong activator of CIC gene expression (Figure 2) [28]. Transfection experiments with pGL3 basic-LUC vector containing the FOXA1 bp region of the CIC gene promoter revealed an increase in LUC activity. Modulation of FOXA1 levels by overexpression and silencing experiments determines a significant increase and decrease in CIC transcript and protein, respectively. FOXA1 belongs to the forkhead box transcription factors, which also include FOXA2 and FOXA3, and which also play a major role in carbohydrate and lipid metabolism and the differentiation of pancreas, liver, lung, and gut [29]. In particular, FOXA1 plays a specific role in hormonal regulation since it induces glucagon gene expression and insulin secretion in pancreatic cells. Some studies highlighted a relationship between insulin secretion and FOXA1 in INS-1 cells [30,31]. Joseph *et al.* [32] demonstrated an involvement of CIC in insulin secretion, and Iacobazzi *et al*. [28] demonstrated the key role performed by FOXA1 gene silencing in the inhibition of glucose-stimulated insulin secretion. In fact, CIC gene silencing, as well as FOXA1 silencing, inhibit glucose-stimulated insulin secretion in INS-1 cells without affecting the cytosolic ATP/ADP ratio. 

Recently, it has been reported that the atypical antipsychotic drug clozapine increases CIC gene expression through up-regulation of FOXA1 in INS-1 and other cells [33]. In INS-1 cells, this effect is only exerted at a basal glucose concentration with a concomitant increase of insulin release. The mechanism of this finding is a matter of debate. Sasaki *et al.* [34] suggested an impairment of the TCA (tricarboxylic acids) cycle in mitochondria through inhibition of clozapine with a subsequent reduction of Krebs cycle metabolites, such as citrate, that act as signals/second messengers in insulin secretion. Interestingly, however, the abnormal insulin secretion under basal conditions is completely abolished by FOXA1 silencing in INS-1 cells treated with clozapine [33].

*SREBP-1a.* An active SREBP-1a (sterol responsive element binding protein) is present in the CIC gene promoter at position –1696/–1686 bp (Figure 2) [35]. SREBP-1a belongs to the basic helix–loop–helix leucine zipper transcription factors, along with SREBP-1c and SREBP-2. These factors regulate transcription of target genes, whose products play key roles in lipid metabolism [36]. In agreement with the reported role performed by SREBPs in the activation of genes involved in fatty acid biosynthesis [37], SREBP-1a activates CIC gene expression in HepG2 cells. It is known that lipogenic enzymes are coordinately regulated at the transcriptional level during starvation and the induction by nutrients [38,39]. Starvation decreases SREBP-1 gene expression and, consequently, the downstream regulated genes [40]. Polyunsaturated fatty acids (PUFAs) (docohexanoic and arachidonic acids) down-regulate CIC gene expression at the transcriptional and translational level. Since both glucose and insulin are required for the production of fatty acids via induction of hepatic lipogenic enzymes, some lines of evidence suggest that the role of glucose and insulin in this action is mediated by induction of SREBP-1c [41,42]. Up-regulation of the CIC gene by insulin is in line with this finding, although a different SREBP isoform, SREBP-1a, is involved. Insulin activates SREBP-1a gene transcription through phosphatidylinositol 3-kinase (PI 3-kinase) and the Akt-dependent pathway. In fact, the increase of CIC expression is abolished by the presence of LY-294002, an inhibitor of PI 3-kinase, which blocks the insulin-induced phosphorylation pathway [43].

*NF-kB.* Two specific binding elements for NF-kB (nuclear factor-kB) are present in the CIC gene promoter at −1314/−1305 bp and −414/−405 bp, respectively [44]. The NF-kB binding site activity was proven in the context of CIC involvement in the inflammation pathway. Infantino *et al.* [44] demonstrated that bacterial lipopolysaccharide (LPS)-induced macrophages greatly activate CIC gene expression through NF-kB, compared to undifferentiated monocytes. Using either siRNA to decrease the amount of CIC or the CIC inhibitor benzene-1,2,3-tricarboxylate (BTA), it was demonstrated that the LPS-induced increase of nitric oxide (NO), ROS and prostaglandins was reduced. These data indicate that CIC is critical for these LPS responses, and the most plausible explanation is that citrate must be transported by CIC into the cytosol for the production of NO, ROS and prostaglandins during inflammation. In fact, an increase of acetyl-CoA derived from CIC-exported citrate is needed to synthesize fatty acids and their derivatives, such as prostaglandins, and to supply NADPH + H^+ ^, which is required for the production of inflammatory mediators, such as NO and ROS. 

This somewhat unexpected role for CIC in LPS action adds to the growing literature on the role of metabolism in the regulation of signaling in inflammation [45]. However, the mechanism by which CIC transcription is regulated in inflammation needs further investigation, because apart from NF-kB, other transcriptional factors may be involved in CIC gene transcriptional activation.

*PPARα/γ.* CIC expression is regulated also by peroxysome proliferator-activated receptor (PPAR) alpha and gamma in rat hepatocytes (BRL-3A) and adipocytes (3T3-L1), respectively [46]. WY-14,643 and rosiglitazone, agonists of PPARα and PPARγ, respectively, greatly increase the expression of CIC. By utilizing LUC activity measurements, gel shift, and ChIP experiments, an active PPRE site was identified in the rat CIC gene promoter at position –625 bp [46]. Moreover, CIC gene expression increases during differentiation of adipocytes, most likely through an involvement of PPARγ.

### 2.2. Repressors

*ZNF224*. By affinity chromatography and mass spectrometry, a zinc finger protein, ZNF224, that binds to the −595/−569 bp sequence in the CIC gene promoter, was identified in HepG2 cells (Figure 2) [47]. This promoter element displays a pronounced repression of CIC transcription, as it strongly decreases LUC transgene expression activity in transfected HepG2 cells. In contrast, ZNF224 silencing markedly activates LUC reporter activity in HEK293 cells transfected with the LUC vector harboring the −595/−569 bp region. Moreover, it exhibits highly specific protein-binding activity in the presence of HepG2 nuclear extracts, as shown by site-directed mutagenesis within the −595/−569 bp sequence. ZNF224 belongs to the Krüppel-like zinc finger family of transcriptional factors that are involved in cell differentiation, cell proliferation, morphogenesis and are expressed during embryonic development [48]. ZNF224 contains a Krüppel-associated box (KRAB) domain, which represses transcription depending on specific interaction with the KAP-1 co-repressor molecule. It was demonstrated that Kap1 binds to KRAB domains as an oligomer, functioning as a scaffold to recruit histone deacetylases [49]. 

The presence of negative regulatory elements in the CIC gene promoter is thought to be important to determine timing of transcription during development. ZNF224 and the CIC gene reveal a different pattern of expression: ZNF224 is present in small amounts in adult tissues and at very high levels in fetal tissues [49], whereas the CIC gene is expressed in small amounts in fetal liver and kidney as compared to the same adult tissues. This different regulation may be related to the fact that, in the fetus, fatty acid biosynthesis occurs at very low levels because the placenta is relatively permeable to free fatty acids, especially PUFAs. Maternal plasma triglycerides are taken up by the placenta where their intracellular hydrolysis facilitates the diffusion of fatty acids to the fetus [50]. By contrast, in advanced gestation, there is a gradual shift to *de novo* synthesis with an increase in CIC expression needed to transport the acetyl-CoA unit as a citrate outside mitochondria for fatty acid synthesis. Moreover, low expression of CIC in fetal tissue is also due to the higher concentration of PUFAs in fetal blood with respect to that of the mother. In fact, the human placenta membrane contains a fatty acid-binding protein, which selectively transports PUFAs [51]. In tissues such as brain, about half of total lipid content is composed of long-chain unsaturated fatty acids, of which arachidonic and docosahexaenoic acids are metabolically more important. PUFAs repress CIC gene expression via the SREBP-1 transcription factor [35]. It is not excluded that the CIC negative regulatory element ZNF224 is part of a more intricate DNA–protein interaction that plays a role in modulating CIC mRNA in fetal life.

### 2.3. Epigenetic Mechanisms Regulating CIC Expression

As mentioned in the description of the role performed by Sp1, CpG methylation is a key factor in regulating CIC gene expression (Figure 2) [26]. Epigenetic regulation was demonstrated on different cell lines, HepG2, HEK293 and SK-N-SH, which express different amounts of CIC in relation to fatty acid biosynthesis activity. In hepatic cell lines, the CIC gene CpG island is hypomethylated and thus associated to Sp1, while histones are acetylated. This finding is based on the observation that CIC expression was affected neither by treatment with 5-Azacitidine (5-AzaC), an inhibitor of DNA methyltransferase, nor by treatment with tricostatin (TSA), an inhibitor of histone deacetylase. A CIC repressed status is present in neuroblastoma cells. ChIP assay revealed that CpG island methylation and H3 histone deacetylation inhibit the binding of Sp1. In these cells, demethylation of promoter due to exposure to 5-AzaC activates expression of CIC by 50%. However, simple demethylation is not sufficient to promote maximal expression, as long as chromatin condensation persists. Maximal induction of expression of the CIC gene in SK-N-SH cells requires the synergistic action of TSA and 5-AzaC, supporting the concept that alteration of the chromatin structure is necessary for the DNA demethylation process. This different regulation most likely reflects the metabolic role of CIC in different cells. In HepG2, CIC plays an important role in fatty acid metabolismand requires constant activation. By contrast, CIC is not so important to the metabolism of neuroblastoma cells and is therefore silenced by the epigenetic mechanism and other mechanisms and factors not yet identifed.

### 2.4. Hormones Regulating CIC Expression

Two hormones are implicated in the control of CIC expression. One such hormone is insulin, an anabolic hormone that controls blood glucose levels and energy homeostasis and is involved in the pathology of metabolic disorders. Studies performed by Kaplan demonstrated that diabetic rats show reduced CIC activity, whereas the general mitochondrial function is not altered [52]. The reduced CIC activity observed in the diabetic state can be ascribed to either transcriptional or post-transcriptional CIC gene regulation [53]. Treatment of diabetic rats with insulin reverses the reduction of CIC activity [54]. In agreement with this finding, insulin increases human CIC gene expression in the HepG2 cell line through involvement of SREBP-1 [35], and CIC plays a regulatory role in glucose-stimulated insulin secretion [32].

Another hormone that influences CIC expression is 3,3’,5-triiodo-L-thyronine (T3) that mantains lipid homeostasis via its effect on gene expression in target organs, mainly in liver and adipose tissue. CIC activity, mRNA and protein are enhanced in hyperthyroidism and reduced significantly during starvation [55]. Change in CIC expression is due to the action of an unidentified *cis*-acting element, most likely located on exon 8, which inhibits the splicing process [56]. In the context of lipid metabolism, T3 increases the expression of several genes involved in hepatic lipogenesis, including fatty acid synthase, acyl-CoA synthase, fatty acid transporter protein, and malic enzyme [57]. T3 also induces the transcription of genes involved in fatty acid oxidation, such as lipoprotein lipase, fatty acid-binding protein [57], and CPT-1 [58].

## 3. CAC Gene Transcriptional Regulation

The CAC gene is located on chromosome 3p21.31 [59]. It spans about 42 kb and is split into nine exons with the translation start site in exon 1 [60]. The CAC gene is differently expressed in human tissues. High levels of transcripts are found in liver, heart and skeletal muscle, where β-oxidation is essential for energy production; much lower levels are observed in other tissues, such as brain, placenta, kidney, pancreas and lung [61]. 

**Figure 3:**
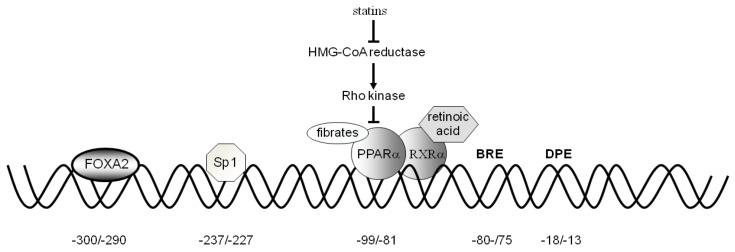
The effect of statins, fibrates and retinoic acid on the PPARα and RXRα-induced CAC gene expression. The binding of fibrates to PPARα, the activation by statins through the Rho-signaling pathway and the binding of retinoic acid to the dimer PPARα-RXRα are shown. The positions of the consensus sequences for FOXA2, Sp1, PPARα, BRE, and DPE in the CAC gene proximal promoter are also indicated.

The human CAC core promoter region revealed the absence of the TATA box element and the presence of the TFB II recognition element (BRE) at −81/−75 bp and of the DPE (AGTGAC) at −18/−13 bp. These elements allow binding of the general transcription factors. The proximal promoter revealed the presence of binding sites for different transcription factors. The following factors, acting as activators, have been functionally tested: PPARα, Sp1, FOXA2, SRC-3, and ERR (Figure 3). 

### 3.1. Activators

*PPARα.* An active binding site for PPARα is present in the CAC gene promoter at position −99/−80 bp (Figure 3) [62]. The promoter activity of the CAC gene PPRE was proven by transfecting HepG2 cells with the pGL3 basic-LUC vector containing the –334/+3 bp region of the CAC gene. Mutations in the PPARα binding sites abolished CAC activation. In agreement with the role performed in the fatty acid catabolism pathway, PPARα is also a strong activator of CAC gene expression. It is known that PPARα., a member of the nuclear receptor super family of ligand-activated transcription factors, has a central role in the regulation of many genes involved in cellular fatty acid uptake and oxidation and provides a molecular link between the major pathways of energy metabolism [63]. The role of PPARα in fatty acid β-oxidation is highlighted by the observation that fasting results in an enhanced load of fatty acids in the liver to be used as an energy source [5].

CAC expression is further increased by hypolipidemic drugs such as statins, fibrates and 9-cis-retinoic acid [62]. HepG2 cells transfected with the wild-type PPRE-containing the pGL3 basic construct and incubated with GW7647 and/or fluvastatin exhibited an increase in LUC activity. Co-treatment of cells with both drugs triggered a synergistic rise in LUC activity by 304%. 9-Cis-retinoic acid increased CAC gene promoter activity only in the presence of wild-type PPRE as compared to control. Combination treatment with GW7647 and 9-cis-retinoic acid resulted in a still greater stimulation of gene reporter activity [62]. These drugs act according to different mechanisms. Fibrates exert their effect by binding to PPARα [64], statins by inhibiting the Rho-signaling pathway [65], and retinoic acid by activating PPRE when bound to the PPARα–RXRα heterodimer [66]. Finally, the PKA pathway also activates CAC gene expression. In HepG2 cells transfected with wild-type PPRE-containing the pGL3 basic construct and treated with forskolin, alone or in combination with GW7647, gene reporter activity was increased by 49% and 138%, respectively. These findings are particularly important because patients affected by CAC deficiency, who present a mild phenotype with some residual activity [67,68,69,70,71], might benefit from treatment with statins and fibrates acting via stimulation of CAC gene expression.

*Sp1 and FOXA2.* The CAC gene proximal promoter also contains two binding sites for Sp1 and FOXA2 at positions −232/−2248 bp and −300/−290 bp, respectively [72]. The role of the FOXA and Sp1 sites in the regulation of CAC gene expression was investigated in HepG2, HEK293 and SK-N-SH cell lines. Transfection of these cells with a construct containing the CAC promoter region from −334/+3 bp upstream of the LUC gene-coding sequence showed that HepG2 cells exhibit a much higher gene reporter activity than HEK293 and SK-N-SH cells. Consistently, HepG2 cells exhibited a higher level of CAC transcript and CAC protein than the other two cell types. ChIP experiments indicated that both FOXA2 and Sp1 in HepG2 cells, and only Sp1 in HEK293 and SK-N-SH cells, are bound to the CAC proximal promoter. This finding indicates that, in the context of fatty acid β-oxidation, Sp1 alone is not sufficient to activate the transcriptional machinery efficiently in liver, and that a liver-specific factor is needed for a high activation of the CAC gene expression. 

FOXA2 is a liver- and pancreas-specific transcriptional factor essential for glucose and lipid homeostasis [73]. Other genes encoding hepatic enzymes involved in metabolism during fasting and energy deprivation are transcriptionally regulated by FOXA2. Among these enzymes are CPT-1, hydroxyacyl-CoA dehydrogenase and lipoprotein lipase (lipid catabolism), phosphoenolpyruvate carboxykinase and glucose-6-phosphatase (gluconeogenesis), and hydroxy-3-methylglutaryl-CoA synthase 1 (ketogenesis) [74]. The presence of FOXA2 in liver could explain, at least in part, the differences in CAC expression levels between liver and other tissues [61]. However, it may be that other transcriptional factors, not yet identified, contribute to an efficient activation of the transcriptional machinery. 

*SRC-3*. Using a comprehensive metabolomics-based analysis, it was found that steroid receptor coactivator-3 (SRC-3) [75], but not SRC-1 and SRC-2, is necessary for the proper transport and metabolism of long-chain fatty acids through a specific regulation of CAC gene expression [76]. The metabolomics analysis was confirmed by transfection experiments. A LUC construct containing the mouse CAC proximal promoter (−995/+133 bp) induced a robust activation of LUC activity in the muscle cell line, C2C12. Furthermore, ChIP performed *in vitro* on C2C12 cells and *in vivo* using skeletal muscle isolated from SRC-3^+/+^ and SRC-3^−/−^ mice demonstrated that SRC-3 is bound to the CAC promoter. Mice lacking SRC-3 show a marked deficiency in CAC expression, which is accompanied by phenotypes that mimic the clinical symptoms of CAC deficiency in humans [77]. Metabolomics analysis of plasma from SRC-3^+/+^ and SRC-3^−/−^ mice demonstrated that SRC-3 knock-out causes an increase in the level of 15 amino acids and an elevation of the urea cycle intermediates ornithine, citrulline, and arginine, together with an elevation of urea cycle enzymes. SRC-3^−/−^ mice also display a variety of cardiac abnormalities. Interestingly, loss of SRC-3 conferred no alterations in 12 other genes essential for fatty acid β-oxidation and ketogenesis, thereby suggesting that CAC is a specific target for SRC-3, at least in mouse muscle. Moreover, SRC-3^−/−^ mice display increased insulin sensitivity, as determined by the hyperinsulinemic/euglycemic clamp, which has been reported in other cases of impaired fatty acid oxidation [78]. 

Because SRC-3 ablation mimics all clinical symptoms of CAC deficiency, it is possible to hypothesize a dysfunctional mutation in the SRC-3 gene in patients who present symptoms of CAC deficiency but harbor no pathogenic mutation in the CAC gene. To test this hypothesis, York *et al.* [76] analyzed 24 patients who exhibited CAC deficient-like symptoms, but failed to show any mutations in the CAC gene. In these patients, analysis of the SRC-3 coding region and flanking intron regions revealed no deleterious mutation, although some patients revealed heterozygous missense variations of unknown clinical significance. Although a number of studies have identified metabolic roles for SRC-3 in glycogenolysis, gluconeogenesis, dietary fat absorption and lipid storage [75], its new role as a key transcriptional activator of the CAC gene may contribute to understanding some unexplained disorders of lipid homeostasis.

*ERR.* An active consensus sequence for the estrogen-related receptor (ERR) is present in the mouse CAC proximal promoter 808 bp upstream of the transcription start site [79]. This sequence binds ERRα both *in vitro* and *in vivo*, as shown by gel shift and ChIP assays. However, ERRα alone is insufficient to activate CAC gene expression since nuclear receptor activity depends on the presence of co-regulatory proteins, such as the members of the PGC-1 co-activator family [80]. In fact, C2C12 myoblast infection with adenovirus expressing ERRα and PGC-1α or 1β showed that CAC gene activation is only evident in the presence of PGC-1α or 1β, co-activators of ERRα. An inverse agonist of ERRα, XTC790, specifically blocks CAC activation by PGC-1β, but not 1α, in C2C12 cells. All these data are consistent with previous results obtained by analyzing the effect of ERRα loss on global gene expression in heart, including genes involved in fatty acid metabolism [81]. In fact, loss of ERRα function causes CAC down-regulation in SAOS2 cells [81], and ERR silencing by siRNA reduces expression of genes up-regulated by PGC-1α, such as CAC [81].

## 4. Regulation of CIC and CAC Expression by Fatty Acids

Fatty acids, PUFAs in particular, are well-characterized nutrients that control and regulate gene expression [82]. The mechanism by which they affect gene expression is complex and involves multiple processes. For example, PUFAs regulate two groups of transcriptional factors, PPAR and SREBP receptors, that are critical for modulating the expression of genes controlling both systemic and tissue-specific lipid homeostasis [83]. While PUFAs activate β-oxidation, they reduce SREBP gene expression and, hence, inhibit expression of related genes. In fact, lipogenic enzymes, such as acetyl-CoA carboxylase and fatty acid synthase, are strongly reduced by a diet enriched with PUFAs of the n-6 or n-3 series [84]. In line with this finding, CIC gene expression is decreased by the presence of PUFAs (docosahexaenoic acid or arachidonic acid), but is unaffected by monounsaturated (oleic acid) or saturated (palmitic acid) fatty acids in HepG2 cells and hepatocytes [35]. Experiments performed using rats fed with PUFA-enriched diets confirm the same specificity [84]. The mechanism of this specificity is not clear. However, a PUFA response region is present in the rat CIC gene promoter [85] and PUFAs cause a reduction of the rate of CIC gene transcription through an inhibition of the splicing process [86].

Interestingly, dietary fat composition also affects CAC activity and expression [87]. PUFAs of the n-3 series increase CAC activity and expression, whereas monounsaturated fatty acids decrease both CAC activity and expression. No effect was observed with PUFAs of the n-6 series and saturated fatty acids. Modulation of CAC expression is ascribed to transcriptional and post-transcriptional events [88]. In fact, real-time and nuclear run-on experiments showed that the transcriptional rate of the CAC gene increases by about 60% in the nuclei of n-3 PUFA-treated rats compared to control. Conversely, post-transcriptional events, most likely concerning CAC mRNA splicing, determined a decrease of CAC gene expression. 

Finally, PUFAs control not only lipid metabolism but also inflammatory factors and cellular events in cardiomyocytes and vascular endothelial cells [87,89]. Because PUFAs suppress the nuclear levels of NF-kB in several model systems [90], it is likely that they control the expression of a subset of NF-kB-regulated genes, including the CIC gene, whose role in inflammation has been recently assessed [35]. 

## 5. Conclusions

After their identification [91,92,93,94,95,96,97], CIC and CAC have been extensively studied using different approaches. Heterologous expression in yeast and *Escherichia coli*, site-directed mutagenesis, reconstitution of the recombinant proteins in phospholipid vesicles, and molecular labeling have allowed the characterization of biochemical properties and structure-function relationships of CIC and CAC in various organisms and tissues [98,99,100,101,102,103,104,105,106,107,108,109,110,111,112,113,114,115]. These studies contributed to elucidating their role as recognized members of the mitochondrial carrier family in fatty acid metabolism. More recently, molecular biology techniques have advanced our understanding of the transcriptional regulation of the CIC and CAC genes. In this review, we have summarized the present knowledge concerning the molecular mechanisms by which the expression of the human CIC and CAC genes are regulated in the context of fatty acid metabolism. The data available confirm the view that fatty acid β-oxidation is mainly controlled by PPARα and, in part, by PGC-1α or 1β, PPARα and ERR activators. Aside from PPARα, CAC gene expression is also regulated by the general factor Sp1, the specific factors FOXA2 and SRC-3, and possibly by other factors not yet identified. Concerning the activation of CAC expression in muscle by SRC-3, but not of other genes essential for β-oxidation and ketogenesis, it remains to be seen whether the activation is tissue specific and whether SRC-3 exerts this effect by interacting with other transcriptional factors. Compared to CAC, the transcriptional regulation of the CIC gene is more diversified, because not only are known lipogenic regulatory factors involved, but also other factors not strictly related to fatty acid metabolism, such as NF-kΒ and FOXA1, thereby indicating that CIC plays a role in other cellular functions and processes besides fatty acid biosynthesis. Recently, evidence has emerged that CIC is a determining factor in tumorigenesis because CIC levels are increased in cancers and its inhibition has anti-tumor activity [116]. The complex interplay between oncogenic signaling and lipid metabolism highlights the importance of a more in-depth understanding of lipid metabolism alterations. CIC is also implicated in LPS-stimulated inflammation [44]. Future research will have to clarify whether pro-inflammatory stimuli such as TNF-α and interleukins up-regulate CIC expression. It will also be interesting to investigate whether the transcriptional activation of CIC is relevant in acute inflammation, which has a beneficial effect against infection and injury, and/or in chronic inflammation, a common feature of various diseases, including neurodegenerative disorders and rheumatoid arthritis. Another interesting aspect of CIC transcriptional regulation, *i.e.*, its activation by PPARα, warrants some comments. The up-regulation of CIC gene expression by PPARα seems contradictory, because PPARα is the master regulator of β-oxidation enzymes. However, several facts must be considered: (i) PPARα responds to changes in both exogenous (dietary) and newly synthesized fat; (ii) certain fatty acids are more effective activators of PPARα than others. For example, monounsaturated oleic acid has no effect on PPARα expression, docosahexaenoic acid is a weak activator whereas eicosapentaenoic acid a strong activator; and (iii) the enzymes involved in the synthesis of PUFAs are regulated by dietary fat. It is likely that the regulation of CIC expression by PPARα is highly controlled by fatty acid cell content. In general, many basic issues remain to be elucidated concerning the functional diversity and the regulatory mechanisms that generate or interconvert different lipids, in addition to the molecular details about how specific fatty acids affect transcriptional factors. 

Although definite progress in the study of the regulation of CIC and CAC gene expression has been achieved in the last few years, not all aspects are completely understood. Full comprehension of the mechanisms regulating CIC and CAC gene expression in different species, tissues, metabolic and hormonal states, and stress conditions will help to better understand the role of CIC and CAC in intermediary metabolism and fatty acid metabolism. Moreover, knowledge of the mechanisms by which different factors control CIC and CAC gene expression may provide insight into the development of new therapeutic strategies for an improved management of whole-body lipid metabolism.
